# The Complex Role of Store Operated Calcium Entry Pathways and Related Proteins in the Function of Cardiac, Skeletal and Vascular Smooth Muscle Cells

**DOI:** 10.3389/fphys.2018.00257

**Published:** 2018-03-21

**Authors:** Javier Avila-Medina, Isabel Mayoral-Gonzalez, Alejandro Dominguez-Rodriguez, Isabel Gallardo-Castillo, Juan Ribas, Antonio Ordoñez, Juan A. Rosado, Tarik Smani

**Affiliations:** ^1^Department of Medical Physiology and Biophysics, University of Seville, Sevilla, Spain; ^2^Institute of Biomedicine of Seville, University Hospital of Virgen del Rocío, CSIC, University of Seville, Sevilla, Spain; ^3^CIBERCV, Madrid, Spain; ^4^Department of Surgery, University of Seville, Sevilla, Spain; ^5^Department of Stomatology, School of Dentistry, University of Seville, Sevilla, Spain; ^6^Cell Physiology Research Group, Department of Physiology, Institute of Molecular Pathology Biomarkers, University of Extremadura, Cáceres, Spain

**Keywords:** Ca^2+^, Orai, STIM, TRPC, cardiomyocyte, skeletal muscle, vascular smooth muscle

## Abstract

Cardiac, skeletal, and smooth muscle cells shared the common feature of contraction in response to different stimuli. Agonist-induced muscle's contraction is triggered by a cytosolic free Ca^2+^ concentration increase due to a rapid Ca^2+^ release from intracellular stores and a transmembrane Ca^2+^ influx, mainly through L-type Ca^2+^ channels. Compelling evidences have demonstrated that Ca^2+^ might also enter through other cationic channels such as Store-Operated Ca^2+^ Channels (SOCCs), involved in several physiological functions and pathological conditions. The opening of SOCCs is regulated by the filling state of the intracellular Ca^2+^ store, the sarcoplasmic reticulum, which communicates to the plasma membrane channels through the Stromal Interaction Molecule 1/2 (STIM1/2) protein. In muscle cells, SOCCs can be mainly non-selective cation channels formed by Orai1 and other members of the Transient Receptor Potential-Canonical (TRPC) channels family, as well as highly selective Ca^2+^ Release-Activated Ca^2+^ (CRAC) channels, formed exclusively by subunits of Orai proteins likely organized in macromolecular complexes. This review summarizes the current knowledge of the complex role of Store Operated Calcium Entry (SOCE) pathways and related proteins in the function of cardiac, skeletal, and vascular smooth muscle cells.

## General overview of store-operated Ca^2+^ entry

Physiological agonists promote a rise in intracellular free Ca^2+^ concentration ([Ca^2+^]_i_) involving Ca^2+^ release from intracellular stores, the Endoplasmic or Sarcoplasmic Reticulum (ER/SR), and Ca^2+^ entry through ion channels located in the Plasma Membrane (PM) (Jackson, 2000). In 1986, Putney suggested that the reduction of internal Ca^2+^ storees could control this PM Ca^2+^ influx. The mechanism was originally named Capacitative Ca^2+^ Entry (CCE) because it was first thought that the Ca^2+^ ions, which enter through PM channels, could be taken up directly into the ER as in a capacitor (Putney, 1986). CCE was later named Store-Operated Ca^2+^ Entry (SOCE) by analogy to voltage-operated Ca^2+^ channels. In 1990 Kwan and colleagues demonstrated that the refilling process does not involve a direct route to the intracellular stores, but it is the result of a sequential Ca^2+^ transport into the cytoplasm and the subsequent uptake from the stores by Sarco/Endoplasmic Reticulum Ca^2+^-ATPase (SERCA) (Kwan et al., 1990). Subsequently, SOCE has been characterized and widely studied in non-excitable cell types (Tojyo et al., 2014; Verkhratsky and Parpura, 2014; Lopez et al., 2017), while its role in excitable cells was difficult to demonstrate. However, few years before the first formulation of Putney's model (Putney, 1986), Casteels and Droogmans reported similar observations in Vascular Smooth Muscle Cells (VSMCs) (Casteels and Droogmans, 1981). Later, compelling evidences established SOCE in neonatal and adult cardiac muscle (Hunton et al., 2002; Uehara et al., 2002; Collins et al., 2013), in different vascular beds (Trepakova et al., 2001; Leung et al., 2008; Park et al., 2008; Wang et al., 2008), as well as in skeletal muscle (Dirksen, 2009; Stiber and Rosenberg, 2011).

Since the identification of SOCE, both the nature of the Store-Operated Ca^2+^ Channels (SOCCs) and its mechanism of activation, this Ca^2+^ influx has been extensively investigated (Parekh and Putney, 2005; Bolotina, 2008; Hogan and Rao, 2015). Different hypotheses have been resumed in: firstly, a conformational coupling between elements in the ER/SR and the PM or secondly, the indirect coupling between the ER/SR and the PM via diffusible messengers (Rosado, 2006). Afterwards and thanks to the advances in molecular biology techniques, arrays study, genes screening, etc., major breakthroughs have been made in understanding SOCE mechanism of activation, which determined an increasing number of the molecular components of SOCE and their cellular organization. In 2005, the Stromal Interaction Molecule-1 (STIM1) was characterized as the ER/SR luminal Ca^2+^ sensor, using an RNA interference-based screen to identify genes involved in Thapsigargin (TG)-evoked Ca^2+^ entry (Roos et al., 2005). Soon after, in 2006 thanks to whole-genome screening of *Drosophila* S2 cells and gene mapping in patients with the hereditary Severe Combined Immunodeficiency (SCID) syndrome, Orai1 was identified as a pore-forming subunit of SOCC (Feske et al., 2006; Vig et al., 2006). In addition, Orai1 homologs, Orai2 and Orai3, have been also demonstrated to be activated by Ca^2+^ store depletion (Lis et al., 2007; Frischauf et al., 2009). Similarly, an alternative spliced long variant of STIM1, STIM1L, has been related to SOCE especially in skeletal and cardiac muscle (Rosado et al., 2016). Meanwhile, the role of STIM2 in SOCE is still under debate, although STIM2 was suggested to interact to its homolog STIM1 (Williams et al., 2001). Independently of its central role in SOCE activation, STIM1 is also activated by diverse stimuli such as oxidation, temperature, hypoxia, acidification, etc. (Hooper et al., 2013). In this review, we will focus on STIM1 and STIM1L role on SOCE since little is known about STIM2 in cardiac, skeletal and VSMCs.

Independent studies showed that SOCE's players could be modulated by phosphorylation. Smyth et al. showed that phosphorylation of STIM1 at Ser486 and Ser668 inactivates SOCE during mitosis (Smyth et al., 2009). In contrast, later study by Pozo-Guisado et al. demonstrated that Extracellular signal-Regulated Kinases 1/2 (ERK1/2) phosphorylates STIM1 at Ser575, Ser608, and Ser62137; and enhances SOCE in HEK293 cells (Pozo-Guisado et al., 2010). Meanwhile, Kawasaki et al. observed that Protein Kinase C (PKC) phosphorylates Orai1 at N-terminal Ser27 and Ser30 which suppress SOCE (Kawasaki et al., 2010).

**Figure 1:**
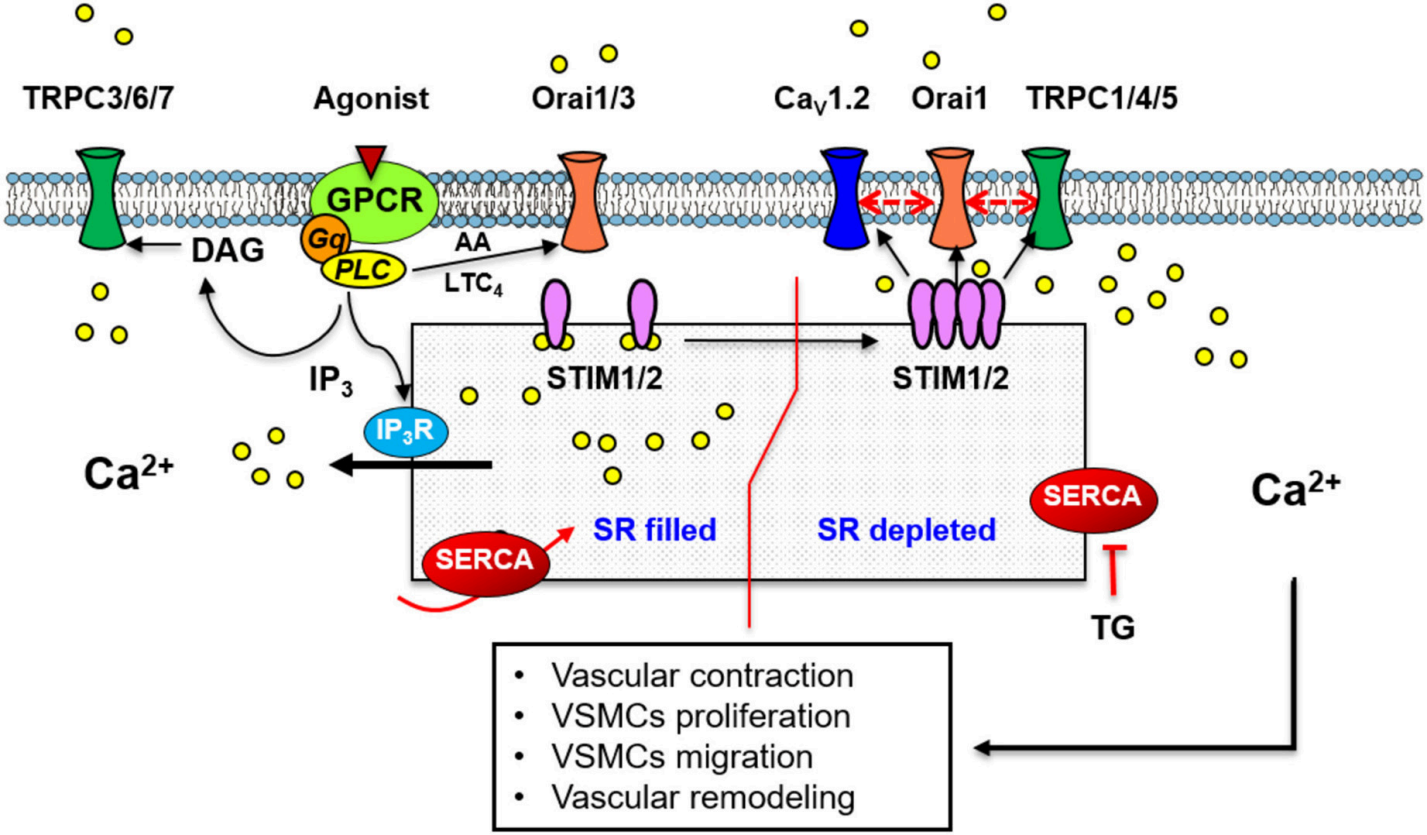
A scheme illustrating the standard mechanism and molecular components of SOCE in vascular smooth muscle cells. Store depletion by thapsigargin or vasoactive agonists binding to GPCR, induces Ca^2+^ store depletion, STIM1 organization in puncta and translocation to the plasma membrane, activation of Ca^2+^ entry through Orai1 and/or TRPC-dependent SOCC. A mechanism involving Orai1, TRPC1 and the voltage-sensitive Cav1.2, has also been implicated in Ca^2+^ influx induced by SOCE. Agonists also stimulate store independent Ca^2+^ entry through Orai1/3 activated by AA or LTC4; or through TRPC3/6/7 activated by DAG.

**Figure 2:**
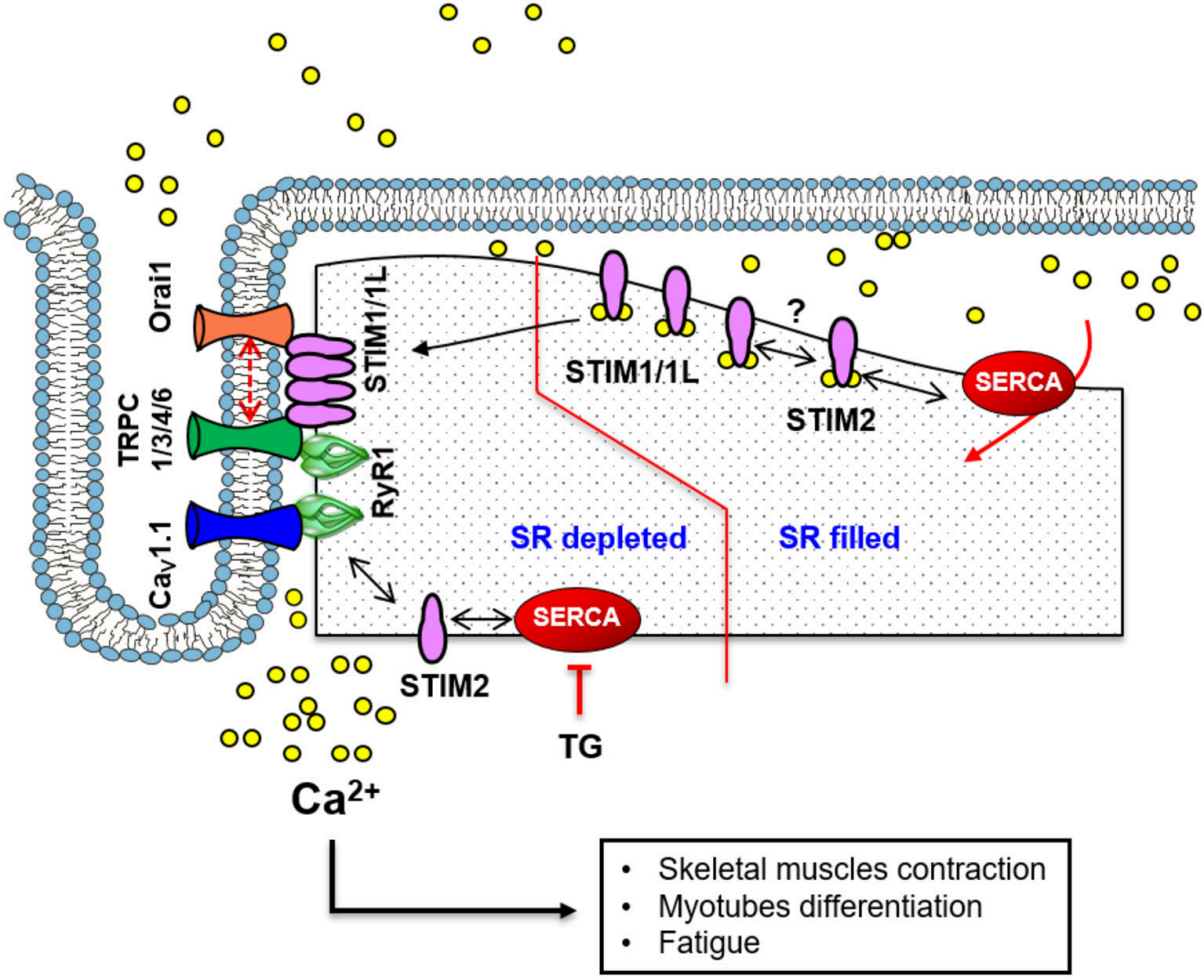
A scheme illustrating the standard mechanism and molecular components of SOCE in skeletal muscle. At the triad junction, the voltage-sensitive Ca_V_1.1 and RyR1 interacts physically. STIM1 and STIM1L are in close proximity or in complex with Orai1 even before store depletion. This pre-localization of STIM1/1L with Orai1 likely facilitates the fast-activation kinetics of SOCE. A similar mechanism involving TRPC channels and STIM1 has also been implicated in Ca^2+^ influx mechanisms. STIM1 interacts with STIM2, which regulates SERCA1-mediated filling of the SR Ca^2+^ store and sustains resting [Ca^2+^] in the cytosol.

**Figure 3:**
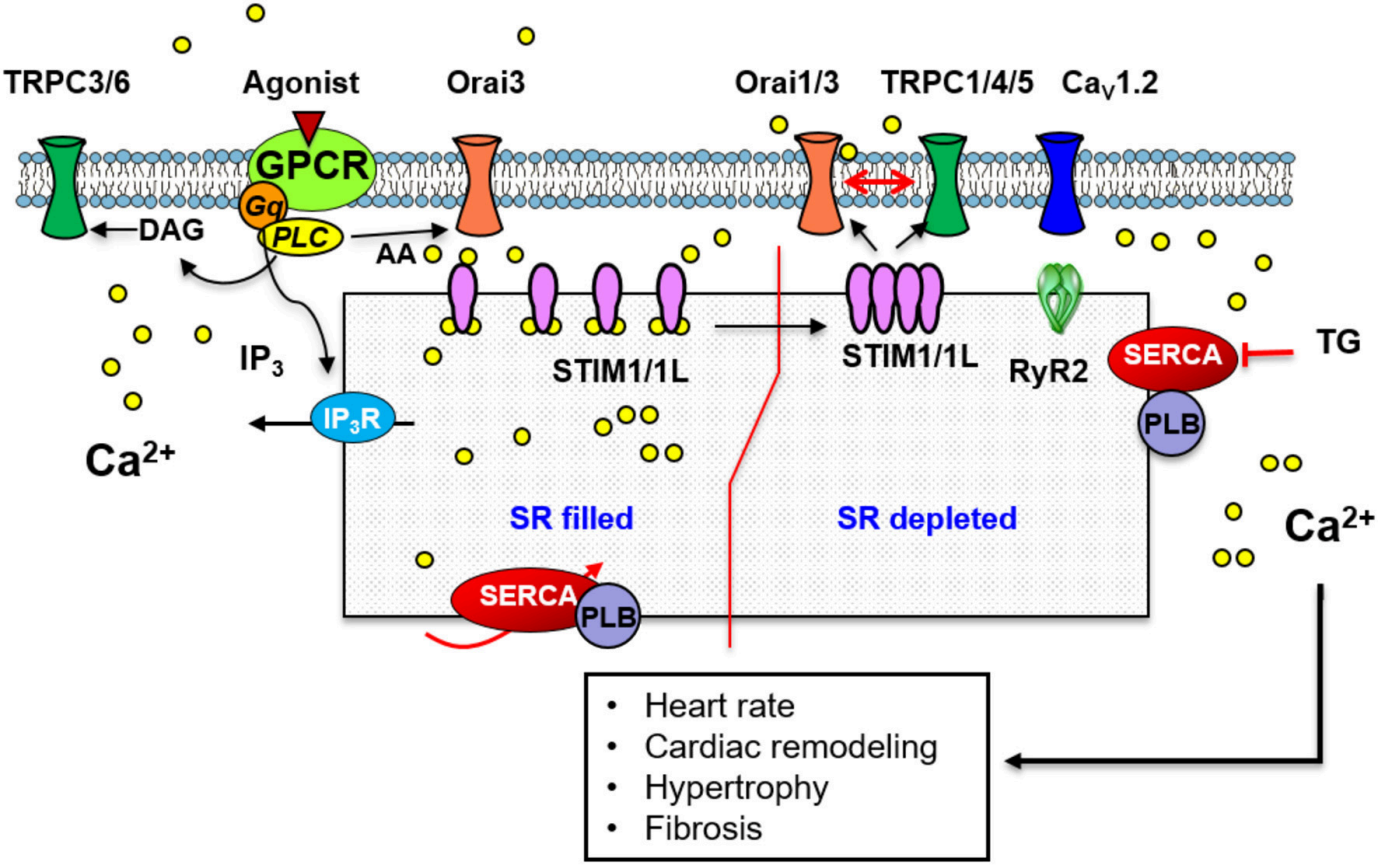
A scheme illustrating the standard mechanism and molecular components of SOCE in cardiac muscle. Store depletion involves STIM1/1L interaction with Orai1 which likely recruits Orai3. STIM1/1L colocalizes with key regulators of SOCE such as Orai1, SERCA, phospholamban (PLB), and RyR2. A similar mechanism involving TRPC1/4/5 channels and STIM1 has also been implicated in SOCE. Store independent Ca^2+^ entry through Orai3 activated by AA, or via TRPC3/6 activated by DAG has been described.

Nowadays, as illustrated in Figures 1–**3**, it is agreed that upon store depletion or Ca^2+^ release from ER/SR, STIM1 moves to distinct punctate aggregates at ER-PM junctions (Liou et al., 2005; Zhang et al., 2005), forming clusters which facilitate the recruitment of Orai1 to the same junctions (Luik et al., 2006; Várnai et al., 2007). In addition, a study from Balla's group suggested the presence of additional molecular components within the STIM1-Orai1 complex, as they found that the colocalization between STIM1 and Orai1 happens only in areas where a larger (12–14 nm) gap exists between the ER and the PM (Várnai et al., 2007). In fact, others molecular components regulating SOCE have been described (Lopez et al., 2016). For example, a protein called Ca^2+^ Release Activated Channel Regulator 2A (CRACR2A), a Ca^2+^ sensor located in the cytoplasm that modulates STIM1–Orai1 complexes (Srikanth et al., 2010); STIM-activating enhancer (STIMATE) an ER-resident protein that apparently modulates the activation and translocation of STIM1 toward ER–PM junctions (Jing et al., 2015); SARAF (SOCE-Associated Regulatory Factor) located in the membrane of the ER which interacts with STIM1 under resting conditions to prevent it spontaneous activation (Palty et al., 2012). Recently, SARAF was also found in the PM in SH-SY5Y neuroblastoma cells where it interacts with Orai1 (Albarran et al., 2016). In addition, lysophospholipids products of Ca^2+^ independent Phospholipase A_2_ (iPLA_2_) were suggested as auxiliary components that co-activate and mediate STIM1–Orai1 interaction (Bolotina, 2008; Smani et al., 2016).

After an intense debate, two types of SOCCs have been proposed so far (Figures 1–**3**): the Ca^2+^ Release-Activated Ca^2+^ (CRAC) channel formed by homo- or hetero-multimeric Orai subunits (Orai1/2) (Desai et al., 2015; Vaeth et al., 2017), and non-selective SOCC involving Orai subunits which interact with members of Transient Receptor Potential Canonical (TRPC) channels (Desai et al., 2015). Actually, many works sustain the involvement of non-selective TRPC subunits in SOCCs (Jardin et al., 2008; Desai et al., 2015). However, the presence of heteromultimers formed by Orai1 and TRPC subunits has not yet proved experimentally. Recently, Ambudkar et al. stated that Ca^2+^ entering through classical Orai1 channels recruits TRPC channels to the PM where they are activated by STIM1 (Ambudkar et al., 2017). In 1992, Hoth and Penner were the first to record in mast cells whole-cell currents activated by Ca^2+^ store depletion, *I*_CRAC_ or Ca^2+^ release-activated Ca^2+^ current. This current is similar to other selective Ca^2+^ currents in being highly selective for Ca^2+^ with a very large positive reversal potential, but being also strongly inwardly rectifying (Hoth and Penner, 1992). Nevertheless, only few reports succeeded to record *I*_CRAC_ in excitable cells as VSMCs (Potier et al., 2009; Rodríguez-Moyano et al., 2013), or in skeletal muscle (Yarotskyy et al., 2013). In contrast, SOCC currents (*I*_SOC_) are non-selective for Ca^2+^ ions with a linear I/V relationship (Wayman et al., 1996). *I*_SOC_ was initially characterized in VSMCs isolated from rabbit portal vein (Albert and Large, 2002), mouse and rabbit aorta (Trepakova et al., 2000; Smani et al., 2004), rat pulmonary artery (Ng and Gurney, 2001) or A7r5 cell line (Brueggemann et al., 2006). *I*_SOC_ with similar biophysics properties was later recorded in cardiac myocytes (Hunton et al., 2004) and skeletal muscle (Ducret et al., 2006).

## SOCE in vascular smooth muscle cells

The molecular mechanism of SOCE in VSMCs still remains intriguing (Figure 1). The first evidence of SOCE presence in VSMCs, as we mentioned previously, relies to Casteels and Droogmans studies (Casteels and Droogmans, 1981). They suggested two pathways for the transport of Ca^2+^ from the extracellular medium to the noradrenalin-sensitive intracellular store, one including a direct route into the store similar to Putney's description (Putney, 1986). Subsequently, several works determined the activation of SOCE in VSMCs either by vasoactive agonists or SERCA inhibition (Jackson, 2000; Albert and Large, 2002). SOCE is essential in VSMCs because it preserves SR Ca^2+^ homeostasis adequately, therefore it makes possible a proper Ca^2+^ signaling (Manjarrés et al., 2010). SOCE is also relevant to different physiological processes as vascular tone regulation (Park et al., 2008; Domínguez-Rodríguez et al., 2012), vasculogenesis and VSMCs proliferation (Barlow et al., 2006; Rodríguez-Moyano et al., 2013). However, at the same time SOCE is involved in vascular disorders as essential and pulmonary hypertension or restenosis (Tanwar et al., 2017).

### STIM and SOCE in vascular smooth muscle

Several reports confirmed the classical mechanism of SOCE activation in these cells and the concept that STIM proteins operate as SR Ca^2+^ sensors for store depletion in VSMCs (Peel et al., 2006; Dietrich et al., 2007; Wang et al., 2008). As illustrated in Figure 1 SOCE activation involves functional STIM1 organization in puncta and their activation of Ca^2+^ influx through SOCCs, as in other tissues (Wang et al., 2008). In VSMCs and upon store depletion, STIM1 binds to Orai1, but also to TRPC1 to allow SOCE (Rodríguez-Moyano et al., 2013). Interestingly, Wang et al. demonstrated that in VSMCs cell line A7r5, upon SOCE activation STIM1 translocates toward PM, where it binds to Orai1 and to Ca_V_1.2 L-type Ca^2+^ channel (LTCC) (Wang et al., 2010). The role of STIM1 in SOCE was also demonstrated by the effect of its downregulation, which inhibits SOCE and the activated current in VSMCs isolated from aorta, pulmonary or coronary arteries (Domínguez-Rodríguez et al., 2012; Ng et al., 2012). Furthermore, STIM1's role in vascular contraction was by vessel transfection with small interfering RNAs (siRNAs) or delivery of antibodies using the chariot delivery technique considered an ideal tool for functional studies as it quickly and efficiently transports peptides and antibodies directly into cells (Giachini et al., 2009; Domínguez-Rodríguez et al., 2012). These studies showed that vessel transfection with STIM1 antibody or siRNA attenuates TG- or agonist-induced vasoconstriction. Other study demonstrated that α1-adrenergic-mediated aortic contraction is significantly reduced in STIM1 knockout (KO) mice, while depolarization-induced contraction is unchanged (Mancarella et al., 2013).

In addition to its role in vascular reactivity, STIM1 is involved in VSMCs proliferation and migration (Potier et al., 2009; Rodríguez-Moyano et al., 2013). STIM1 upregulation is associated with VSMCs growth, actually the expression of STIM1 is increased in the tunica media of the aorta in spontaneously hypertensive rats (Giachini et al., 2009), and of the carotid artery after balloon injury (Zhang et al., 2011). In contrast, silencing of STIM1 reduces the neointima formation and inhibits the activation of transcription factors as cAMP Response Element Binding protein (CREB) or Nuclear Factor of Activated T-cells (NFAT), associated with VSMCs proliferation (Potier et al., 2009; Rodríguez-Moyano et al., 2013).

Unlike STIM1, the role of STIM2 in SOCE is still unclear. Song and colleagues suggested that STIM2 contributes to SOCE in Pulmonary Artery Smooth Muscle Cells (PASMCs) from Patients with idiopathic Pulmonary Arterial Hypertension (iPAH). STIM2 might play a key role, but is not sufficient, in the upregulation of SOCE and PASMCs proliferation during iPAH (Song et al., 2011). A more recent study from the same group suggest that STIM2 upregulation is necessary to increase the resting cytoplasmic Ca^2+^ levels in patients with iPAH, which activate CREB, NFAT, AKT, and STAT3 signaling pathways that promote cell proliferation (Song et al., 2018). In this way, Fernandez et al. determined that STIM2 upregulation contributes to the transition of PASMCs from a contractile to proliferative phenotype in iPAH patients (Fernandez et al., 2015).

### Orai and SOCE in vascular smooth muscle

In VSMCs, Orai proteins are present and function in the same manner as in other tissues (Beech, 2012). Expression of Orai isoforms, Orai1/2/3, is relatively low in contractile VSMCs; however, their expression is dramatically increased in cultured VSMCs. VSMCs in culture are known to experiment a phenotypic switch to a synthetic or proliferative state, what is correlated with a prominent Ca^2+^ influx that can be dependent or independent of store depletion (Berra-Romani et al., 2008; Bisaillon et al., 2010; Gonzalez-Cobos et al., 2013).

The role of Orai1 in VSMCs processes has been largely studied. Knockdown of Orai1, but not Orai2 or Orai3, inhibits SOCE and *I*_CRAC_ in aortic VSMCs (Potier et al., 2009). Meanwhile, the overexpression of Orai1 recovers SOCE after Orai1 knockdown by siRNA in VSMCs isolated from human saphenous veins (Li et al., 2011). Silencing of Orai1 or its functional inhibition with monoclonal antibodies attenuate significantly SOCE and inhibit agonists-induced, coronary and aorta contraction (Giachini et al., 2009; Domínguez-Rodríguez et al., 2012). Furthermore, different studies demonstrated that Orai1-dependent SOCE is necessary for the proliferation and/or migration of aortic and coronary VSMCs (Potier et al., 2009; König et al., 2013; Rodríguez-Moyano et al., 2013). Orai1 is upregulated in VSMCs during vascular injury or in neointima formation following balloon injury of rat's carotids (Zhang et al., 2011). Orai1 is also required for the activation of NFAT and CREB involved in VSMCs proliferation (Potier et al., 2009; Rodríguez-Moyano et al., 2013).

In addition to the association of Orai1 with others isoform, there are evidences in non-excitable cells demonstrating associations between Orai1 and other channels, such as TRPC1. Orai1 resides in close proximity to TRPC1 channels, which allows them to interact at least functionally as demonstrated by independent reports (Gueguinou et al., 2016; Ambudkar et al., 2017). Using co-immunoprecipitation experiments, our group demonstrated that passive store depletion with TG promotes TRPC1 association with Orai1 as well as STIM1 with Orai1 in aortic rat VSMCs (Rodríguez-Moyano et al., 2013). Similar finding was observed in mouse PASMCs under acute hypoxia (Ng et al., 2012). Recently, we determined that Orai1 interacts with TRPC1 and LTCC, Ca_V_1.2 isoform, to form a macromolecular signaling complex to regulate vascular tone in mice aorta (Avila-Medina et al., 2016) (Figure 1). Likewise, Orai1 also interacts with other ion channels as the SK3 channel activated by Ca^2+^ to regulate agonist-induced contraction in VSMCs (Song et al., 2015), or even with the Ca^2+^-activated big K^+^ (BK) channel to reduce agonist-induced membrane depolarization, preventing excessive contraction of VSMCs (Kwan et al., 2009).

While there is no doubt regarding the role of Orai1 in SOCE, the participation of Orai2 and Orai3 is still unclear. The role of Orai2 in SOCE was described in synthetic proliferating PASMCs (Fernandez et al., 2015). Meanwhile, a recent study suggested that the 3 isoforms of Orai participate in chronic hypoxia-induced elevation of SOCE in PASMCs (Wang et al., 2017). In contrast, silencing of Orai2 or Orai3 barely affects PDGF-induced SOCE and aortic VSMCs migration, meanwhile the knockdown of Orai1 attenuates Ca^2+^ entry and VSMCs migration mediated by PDGF (Bisaillon et al., 2010). Other study proposed a significant role for Orai3 in regulating basal Ca^2+^ levels of SR or in mediating Ca^2+^ release from intracellular stores in human airway smooth muscle cells (Peel et al., 2008). Moreover, Orai3 is widely accepted as an important component of store-independent Arachidonate-Regulated Ca^2+^ (ARC) entry (Mignen et al., 2008), and of store-independent Leukotriene C4 (LTC_4_)-regulated Ca^2+^ entry in VSMCs isolated from aorta (Gonzalez-Cobos et al., 2013).

### TRPC and SOCE in vascular smooth muscle

Although, it still remains a controversial issue, there is a body of evidence supporting the participation of TRPC channels in SOCE in VSMCs (DeHaven et al., 2009; Alonso-Carbajo et al., 2017). Since the discovery of SOCE, contribution of TRP channels, especially TRPC1-7 subfamily, was proposed to form non-selective SOCCs, both in non-excitable and excitable cells (Clapham et al., 2001). Numerous TRPC channels contribute to regulation of membrane potential and vascular tone due, in part, to their Ca^2+^ permeability (Mulier et al., 2017). TRPC channels are not addressed in detail here, but a brief discussion of their role in SOCE, especially of TRPC1, is included.

TRPC1 contribution to SOCE has been intensively investigated in VSMCs (Beech et al., 2003; Leung et al., 2008). Beech was the first to propose TRPC1 as a native SOCC in several vascular territories (Xu and Beech, 2001). Subsequently, others studies using neutralizing antibodies or knockdown mediated by siRNAs of TRPC1 showed partial suppression of SOCE in aorta, pulmonary, portal veins or in cerebral VSMCs (Brueggemann et al., 2006; Li et al., 2008; Ng et al., 2012; Rodríguez-Moyano et al., 2013). TRPC1 silencing also inhibited CREB activation and VSMCs proliferation (Rodríguez-Moyano et al., 2013). In contrast, other studies did not observe a role of TRPC1 in the induced SOCE in synthetic VSMCs or in TRPC1 KO mice (Dietrich et al., 2007; Potier et al., 2009). Recently, a well-designed work from Albert and co-authors showed that in contractile VSMCs, store depletion causes movement of the SR membrane containing activated STIM1 toward the PM, where it interacts with TRPC1, stimulating PLCβ1 and inducing channel opening through DAG-mediated PKC phosphorylation of TRPC1 (Shi et al., 2017). This study might help to clarify the role of TRPC1 in SOCE as previous reports suggested that TRPC1-mediated currents are activated secondarily as a result of PLC activation but not due to store depletion (Zarayskiy et al., 2007). Recently, Ambudkar et al. discussed the role of TRPC channels in SOCE and concluded that Ca^2+^ entering through classical Orai1 channels recruits TRPC1 channels to the PM where they are activated by STIM1 (Ambudkar et al., 2017).

The participation of other TRPC isoforms in SOCE in VSMCs is still controversial. Independent works suggested that TRPC1 forms heteromeric channels with TRPC4 and/or TRPC5 to mediate SOCE in contractile VSMCs (Clapham et al., 2001; Sabourin et al., 2009; Shi et al., 2012). TRPC3 barely participates in SOCE (Thebault et al., 2005), while TRPC4 seems involved in SOCCs in aortic and mesenteric VSMCs (Lindsey et al., 2008). TRPC5 is less expressed in VSMCs and its function is still unknown (Zholos, 2014). Meanwhile, TRPC6 and TRPC7 are mainly considered receptor-operated channels rather than SOCCs (Dietrich et al., 2005; Maruyama et al., 2006; Albert et al., 2008).

## SOCE in skeletal muscle

Intracellular Ca^2+^ is a central player in Excitation-Contraction (EC) coupling to fulfill skeletal muscle's contraction (Ríos et al., 1992). In 2001, a first study showed that repeated application of high concentration of K^+^ combined with SERCA inhibitor (in the absence of external Ca^2+^) induced the depletion of SR which activates SOCE in isolated muscle fibers from adult mice (Kurebayashi and Ogawa, 2001). Subsequent studies further highlighted the presence of Ca^2+^ entry pathways independent of the dihydropyridine receptor in the skeletal muscle and their physiological relevance. Therefore, increasing evidences revealed the importance of SOCE in skeletal muscle contraction, development, fatigue and disease (Dirksen, 2009; Stiber and Rosenberg, 2011; Wei-Lapierre et al., 2013). Remarkably, only few groups succeeded to measure *I*_CRAC_-like and *I*_SOC_ currents in skeletal myotubes (Ducret et al., 2006; Stiber et al., 2008; Yarotskyy and Dirksen, 2012).

SOCE was described in primary cultured myotubes and in adult muscle fibers, in response to depletion of SR Ca^2+^ stores either by inhibition of SERCA with TG or by inhibition of Ryanodine Receptors (RyRs) (Kurebayashi and Ogawa, 2001; Pan et al., 2002, 2014). Currently, the SOCE machinery is believed as a signaling complex located at the triad of skeletal muscle as illustrated in Figure 2. It contains several components, e.g., STIM1, Orai1 and RyRs, which control Ca^2+^ influx to refill SR Ca^2+^ store (Stiber et al., 2008; Pan et al., 2014). However, loss of SOCE does not directly affect EC coupling. In contrast, high frequency stimulation of muscle activates SOCE to sustain muscle contraction (Allen et al., 2008; Pan et al., 2014). Therefore, SOCE likely participates in the muscle contractions during tetanic stimulations in vigorous exercise and fatigue as well as under usual conditions where a contraction might need to be sustained for a long period (Allen et al., 2008). Nevertheless, a recent study suggested that SOCE has a role in maintaining and refilling SR Ca^2+^ stores, not only in repetitive tetanic stimulation, but also on an immediate basis (Sztretye et al., 2017).

Different models for SOCE in skeletal muscle have been proposed (Figure 2), including the conformation coupling between STIM1 and Orai1, STIM1 and TRPC, and RyRs and TRPCs (Kiviluoto et al., 2011). Actually, SOCC might be formed by Orai1 channels in close association with TRPC1/3 or 6 channels to increase the Ca^2+^ entrance (Lyfenko and Dirksen, 2008; Stiber et al., 2008). A particular feature of SOCE in skeletal muscle is its very fast kinetics of activation. The process leading to SOCE (STIM1 aggregation, ER translocation to the PM and Orai1 opening), takes place in less than a second accordingly to some studies (Launikonis et al., 2009; Edwards et al., 2010b). This feature is apparently due to the architecture of the SR that is permanently close to T-tubules at the triad, which allows STIM1 and Orai1 proximity even before store depletion.

### STIM and SOCE in skeletal muscle

Since the identification of STIM1 and Orai1 as components of SOCE, independent studies confirmed that STIM1 is highly expressed in myotubes and adult skeletal muscle (Lyfenko and Dirksen, 2008; Stiber et al., 2008; Darbellay et al., 2010). As in other cell types, STIM1 activates Orai1 to introduce Ca^2+^ inside skeletal muscle (Lyfenko and Dirksen, 2008; Shin and Muallem, 2008). In addition, STIM1 might bind directly to Orai1, to TRPC1/4/5, or indirectly to TRPC3/6 to promote SOCE (Yuan et al., 2007). The importance of STIM1 in SOCE skeletal muscle was clarified by experiments of upregulation and downregulation. STIM1 knockdown by siRNA inhibits SOCE and prevents the refilling of internal Ca^2+^ store (Lyfenko and Dirksen, 2008). Moreover, the integrity of STIM1 turn to be essential for normal development of muscle mass and fatigue (Kiviluoto et al., 2011; Carrell et al., 2016). For example, silencing of STIM1 reduces SOCE and impairs myoblast differentiation, whereas overexpression of STIM1 increases significantly SOCE (Antigny et al., 2013), and accelerates myoblasts' differentiation (Darbellay et al., 2009). Experiments using conditional skeletal muscle STIM1 KO mice also demonstrated that STIM1-mediated SOCE is required for neonatal skeletal growth and differentiation (Li et al., 2012). These KO mice showed an alteration in the maintaining and refilling of SR Ca^2+^ store, a reduction of body mass and susceptibility to fatigue (Li et al., 2012).

The alteration in the expression of STIM1 is also associated with different muscle diseases (Lacruz and Feske, 2015). Patients with SCID, associated with mutations of STIM1 and Orai1 genes, have depressed SOCE, manifest atrophy in skeletal-muscle fibers, amyopathy and a severe chronic pulmonary problem due to respiratory muscle weakness (Kiviluoto et al., 2011; Lacruz and Feske, 2015). Meanwhile, Duchenne Muscular Dystrophy (DMD) mice (*mdx* animal model), have alterations in Ca^2+^-handling proteins involving STIM1 upregulation (Edwards et al., 2010a; Onopiuk et al., 2015). Recently, mice lacking of myostatin gene, which negatively regulates skeletal muscle growth and mass, exhibit reduced STIM1 and Orai1 expression compared to wild type mice. The lower expression of STIM1 and Orai1 in these mice is accompanied by a reduction in SOCE, SR Ca^2+^ content and depolarization-evoked Ca^2+^ release (Sztretye et al., 2017).

In addition, a splice variant of STIM1, called STIM1L, has been described in human skeletal muscle (Horinouchi et al., 2012) and differentiated myotubes (Darbellay et al., 2011; Sztretye et al., 2017). STIM1L contains an extra 106 amino acids insert in the cytosolic region, which provides STIM1 the ability to interact with Orai1 channels; leading to the formation of permanent STIM1L-Orai1 clusters. These clusters are responsible for the rapid (<1 s) activation of SOCE in skeletal muscle in comparison with other cells (Rosado et al., 2016). STIM1L is colocalized with Orai1 as well as others channels, such as TRPC1, TRPC3, TRPC4, and TRPC6 (Horinouchi et al., 2012; Antigny et al., 2017; Saüc and Frieden, 2017). STIM1L silencing evokes significant delay in SOCE activation and promotes the formation of small myotubes. In contrast, STIM1L overexpression accelerates SOCE activation and triggers the formation of larger myotubes (Darbellay et al., 2011; Horinouchi et al., 2012; Antigny et al., 2017; Saüc and Frieden, 2017).

STIM2 is also expressed in myotubes and adult skeletal muscle, and is considered a necessary partner of STIM1 during EC coupling (Darbellay et al., 2010). However, its role in SOCE modulation is still unclear. STIM2 silencing was associated with a reduction in SERCA1a as well as RyR1 activity and SOCE during skeletal muscle contraction (Darbellay et al., 2010; Oh et al., 2017). Other authors have described that STIM2 knockdown inhibits SOCE and C2C12 myoblast differentiation (Phuong and Kang, 2015). STIM2 was proposed to interact with STIM1 to control SOCE during human myoblast differentiation (Darbellay et al., 2010). Interestingly, a recent study demonstrated that STIM2 attenuates SERCA1a activity, which also contributes to the Ca^2+^ distribution between the cytosol and the SR (Oh et al., 2017). This recent report suggested that STIM2 knockdown enhances Ca^2+^ uptake into SR and decreases SOCE in skeletal muscle.

### Orai role in skeletal muscle SOCE

Orai1 is confirmed as the most important isoform in skeletal muscle, meanwhile the role of Orai2 and Orai3 in Ca^2+^ homeostasis is less characterized (Roberts-Thomson et al., 2010). Orai1 is abundantly expressed in neonatal myotubes and adult muscle fibers (Stiber et al., 2008; Dirksen, 2009; Sztretye et al., 2017), and is required for the activation of SOCE in myotubes since the expression of human dominant-negative Orai1 abolishes SOCE (Wei-Lapierre et al., 2013). Upon store depletion endogenous Orai1 colocalizes with STIM1 or STIM1L at triad junction to form STIM1/1L–Orai1 complexes. This colocalization happens even when the SR is full to form permanent clusters at the triad junction responsible of the fast activation of SOCE (Darbellay et al., 2011; Pan et al., 2014).

Experiments using Orai1 KO mice showed diverse results. Some data confirmed a dysregulation in SOCE, in muscle development, muscle mass in adulthood and muscle proper functioning. However, it is not clear whether these effects are related directly with SOCE (Kiviluoto et al., 2011; Wei-Lapierre et al., 2013; Carrell et al., 2016). A recent study using inducible and constitutive muscle-specific Orai1 KO mice showed that activation of Orai1 during muscle use is not required, at least, for maximal performance (Carrell et al., 2016). In contrast, Orai1 mutation R91W, in SCID patients, evokes depressed SOCE and muscular hypotonia (Feske et al., 2006). Others patients with Orai1 A103E/L194P mutations showed similar muscle weakness and hypotonia (Lacruz and Feske, 2015). A recent study by Böhm et al. identified three Orai1 mutations, G98S, V107M and T184M, involved in tubular aggregate myopathy associated with an enhanced SOCE (Böhm et al., 2017). This study suggested that these Orai1 mutations produce constitutively active channels, permeable of Ca^2+^ even in the absence of store depletion. Recently, Sztretye et al. showed that reduced expression of Orai1, together with STIM1 and STIM1L, correlates with a reduction in SOCE, SR calcium content and depolarization-evoked calcium release in mice with myostatin mutation (Sztretye et al., 2017). In contrast, Orai1 is overexpressed in the adult dystrophic muscles in *mdx* mice, which is associated with exacerbated SOCE and SR Ca^2+^ storage (Zhao et al., 2012).

### TRPC channels and SOCE in skeletal muscle

In skeletal muscle, several isoforms of the TRPC subfamily are expressed (Saüc and Frieden, 2017). In particular, TRPC1, TRPC3, TRPC4, and TRPC6 have been consistently found in cultured myoblasts or in adult muscles (Gailly, 2012; Antigny et al., 2013). Most studies have focused on TRPC1 and TRPC4 role in SOCE. TRPC1 gene encodes both a stretch-activated channel, as well as a SOCC (Maroto et al., 2005). TG-induced store depletion activates TRPC1 and TRPC4 (Ducret et al., 2006). Moreover, upregulation of TRPC1 and TRPC4 enhances the Ca^2+^ influx (Sabourin et al., 2009). In contrast, siRNA or dominant negative constructions reduces TRPC1- and TRPC4-dependent SOCE in myoblasts (Antigny et al., 2013). TRPC1 and TRPC4 downregulation also decreases the occurrence of SOCE and affects the formation of normal-sized myotubes (Vandebrouck et al., 2002). Importantly, TRPC1 and/or TRPC4 inhibition recovers Ca^2+^ homeostasis in skeletal muscle fibers from *mdx* mice (Vandebrouck et al., 2002; Kiviluoto et al., 2011).

As reviewed by Kiviluoto and colleagues, in skeletal muscle TRPC likely forms ternary complexes with STIM and Orai1 proteins (Kiviluoto et al., 2011), they might associate only with Orai1 (Stiber and Rosenberg, 2011), or they can even associate to RyRs apparently to trigger SOCE response (Lee et al., 2006). Interestingly similar interaction between TRPC and RyRs has been described earlier in cardiac muscle (Goel et al., 2007) and recently in smooth muscle (Lin et al., 2016). Nevertheless, RyRs are not considered essential for SOCE in skeletal muscle and their conformational coupling with Orai1 or TRPC is no longer accepted, since myotubes of mice lacking RyR1/RyR3 still display prominent SOCE (Zhao et al., 2006; Lyfenko and Dirksen, 2008).

## SOCE in cardiac muscle

Cardiac EC coupling is based on a tightly regulated cascade of Ca^2+^ fluxes. Ca^2+^ entry to the cytosol occurs through LTCC or from SR via RyR release. Meanwhile, its reduction in the cytosol is due to the reuptake of Ca^2+^ by SERCA in the SR or the activity of Na^+^/Ca^2+^ exchanger (NCX) located in the PM (Bers, 2002). The presence of SOCE in cardiomyocytes has been initially ignored since cardiomyocytes have large Ca^2+^ influx arising with each heartbeat (Collins et al., 2013; Bootman and Rietdorf, 2017). Actually, the contribution of SOCE to normal cardiac physiology is still under debate (Bootman and Rietdorf, 2017). However, growing set of experiments have demonstrated the presence of SOCE especially in neonatal cardiomyocytes (Bartoli and Sabourin, 2017). SOCE occurs as a result of an upregulation of ion channels in the course of the reactivated fetal gene program, or because of an increased ion channel activity during chronic cardiac disease development (Eder and Molkentin, 2011; Hulot et al., 2011; Luo et al., 2012). Different works showed that cardiomyocytes stimulation with TG or agonists activating G Protein Coupled Receptors (GPCRs), evokes persistent Ca^2+^ influx that is insensitive to LTCC or NCX inhibition, but sensitive to blockers of SOCE (Uehara et al., 2002; Hunton et al., 2004; Kojima et al., 2012). Store depletion also promotes significant rise in Ca^2+^ entry in pacemaker cells isolated from mouse sinoatrial node, meanwhile SOCE blockers reduce the amplitude and frequency of spontaneous Ca^2+^ transients and decrease Ca^2+^ store content (Liu et al., 2015).

As illustrated in Figure 3, the standard molecular components of SOCE (e.g., Orai, and TRPC proteins) are expressed in adult, neonatal and cell line of cardiomyocytes where they participate in cardiomyocyte development, homeostasis, and genes transcription (Ohba et al., 2007; Voelkers et al., 2010; Hulot et al., 2011; Völkers et al., 2012; Zhu-Mauldin et al., 2012). Independent studies indicate that STIM, Orai, and TRPC proteins contribute to disease conditions such as pathological hypertrophy or conduction disorders (Eder and Molkentin, 2011; Dionisio et al., 2015).

### STIM role in cardiac myocytes

Cardiomyocytes express three isoforms of STIM: STIM1, STIM1L, and STIM2 (Horton et al., 2014; Correll et al., 2015; Zhao et al., 2015), although, little is known regarding the potential role of STIM2 in the heart. STIM1 and STIM1L colocalize with key regulators of SOCE such as SERCA, phospholamban and RyRs in neonatal and adult rat hearts (Zhu-Mauldin et al., 2012; Correll et al., 2015; Zhao et al., 2015). In neonatal cardiomyocytes, SR Ca^2+^ depletion leads to the formation of STIM1 puncta (Hulot et al., 2011) and their interaction with Orai1 (Zhu-Mauldin et al., 2012; Sabourin et al., 2016). Other studies have demonstrated that Ca^2+^ influx observed upon re-addition of extracellular Ca^2+^ could be decreased by downregulation of STIM1 or by its inhibition with dominant-negative mutants (Hulot et al., 2011; Luo et al., 2012). STIM1 siRNA also decreases the frequency of spontaneous Ca^2+^ transients, which affects cytosolic and SR Ca^2+^ handling leading to lower diastolic Ca^2+^ levels and minor SR Ca^2+^ content in neonatal cardiomyocytes (Voelkers et al., 2010). Furthermore, in sinoatrial node STIM1 activates Ca^2+^ entry via Orai1 channels and modifies the heart rate. Cardiac-specific deletion of STIM1 in mice depletes SR Ca^2+^ stores of sinoatrial node cardiomyoctes and leads to pacemaker dysfunction, as was evident by a reduction in heart rate, sinus arrest and an exaggerated autonomic response to cholinergic signaling (Zhang H. et al., 2015). In contrast, a recent study by Zhao and colleagues suggested that STIM1 is not necessary for SOCE activation in cardiomyocytes. They failed to observe SOCE in control or in ventricular myocytes overexpressing STIM1 (Zhao et al., 2015). Moreover, Parks et al. demonstrated that STIM1 forms pre-constituted “punctate” structures without the need of Ca^2+^ store depletion in neonatal and adult cardiomyocytes, the same as in skeletal muscle (Parks et al., 2016). Therefore, the precise role of STIM1 in cardiomyocytes is still unclear and additional studies are needed to clarify the controversy of STIM1 role in SOCE in heart.

In addition to STIM1 role in cardiac homeostasis, compelling evidences characterized the role of STIM1-mediated SOCE in cardiac hypertrophy. STIM1 is considered an essential activator of the NFAT transcription factor, a well-known positive regulator of cardiac growth (Heineke and Molkentin, 2006). In agreement with an early finding by Pang et al. who demonstrated that Ca^2+^ influx induced by angiotensin II or SERCA inhibition activates nuclear translocation of NFAT in cardiomyocytes (Pang et al., 2002). Luo and colleagues found that STIM1 levels increase substantially in response to transaortic constriction-induced hypertrophy (Luo et al., 2012). Hulot et al. reported that STIM1 dependent-SOCE is significantly increased following cardiac hypertrophy both *in vitro* and in the adult heart (Hulot et al., 2011). They observed that transfection of cardiomyocytes with constitutively active-STIM1 is sufficient to increase hypertrophy and protein synthesis. In contrast, *in vivo* STIM1 gene silencing protects the heart from pressure overload-induced hypertrophy. Other report showed that STIM1-KO hearts exhibits ER dilatation, disorganization of mitochondrial size and as consequence the development of a dilated cardiomyopathy, revealing an essential role of STIM1 for normal cardiomyocyte function (Collins et al., 2014). A more recent study showed that cardiac STIM1 silencing using AAV9 inhibits cardiac hypertrophy (Bénard et al., 2016). However, these effects in transfected heart or STIM1-KO mice are not necessarily dependent on SOCE. Indeed and independently of its role in SOCE, STIM1 likely participates to the contractile rhythmicity of cardiomyocytes by moderating T-type Ca^2+^ channel expression and activity (Nguyen et al., 2013), increases Ca^2+^ spark rates and induces spontaneous action potentials (Troupes et al., 2017), and its overexpression leads to Ca^2+^ transients dysregulation (Correll et al., 2015; Zhao et al., 2015).

### Orai and SOCE in cardiac myocytes

The three members of the Orai family (Orai1, Orai2, and Orai3) are present in neonatal and adult cardiomyocytes (Collins et al., 2013; Domínguez-Rodríguez et al., 2015; Sabourin et al., 2016), sinoatrial node (Ju et al., 2015; Zhang H. et al., 2015) and/or atrial cells (Touchberry et al., 2011; Wolkowicz et al., 2011). The physio/pathological role of Orai1 in heart has been extensively studied; meanwhile the roles of Orai2 and Orai3 are less defined. Several data demonstrated that Orai1 participates actively in SOCE and might be implicated in the development of diseases such as arrhythmias, cardiac fibrosis or hypertrophy (Ruhle and Trebak, 2013). Previous studies demonstrated that knockdown of Orai1 inhibits significantly TG-induced SOCE, as well as both cytosolic and SR Ca^2+^ levels in neonatal cardiomyocytes and HL-1 cell line (Voelkers et al., 2010; Touchberry et al., 2011). A recent study demonstrated that specific inhibition of Orai1 by Synta66 prevents completely aldosterone evoked diastolic Ca^2+^ overload in neonatal cardiomyocytes (Sabourin et al., 2016). Other recent works suggested that SOCE mediated by Orai1 interaction with STIM1 modifies heart rate by pacemaker stimulation (Zhang H. et al., 2015), and plays a role in the initiation of arrhythmias in both atrial and ventricular myocytes (Wolkowicz et al., 2011; Wang et al., 2012).

As stated before, several studies proposed a pathological key role for STIM1/Orai1-mediated SOCE in altered Ca^2+^ signaling underlying the development of cardiac hypertrophy by changing the fetal gene program regulated by NFAT signaling. Silencing of Orai1 completely revokes phenylephrine-mediated hypertrophic neonatal cardiomyocyte growth, involving NFAT activation (Voelkers et al., 2010). In contrast, loss of Orai1 in KO mice promotes tissue fibrosis and cardiomyocyte apoptosis, which accelerates the dilated cardiomyopathy and heart failure (Horton et al., 2014). Interestingly, a recent study by Saliba et al. demonstrated that Orai3 is recruited to STIM1/Orai1 complexes during cardiac hypertrophy. They observed that Orai1 and Orai3 knockdown prevents SOCE, and Orai3 also drives store-independent Ca^2+^ entry through ARC channel. Therefore, authors suggested that Orai3 interacts with Orai1 and STIM1 to form a signaling complex which is thought necessary for an amplified Ca^2+^ entry in hypertrophied cardiomyocytes (Saliba et al., 2015).

Altogether, these studies confirm that Orai1, and perhaps Orai3, are important regulators of myocardial electromechanical activity, although further investigations are required to determine the precise role of Orai1/3 in cardiac process.

### TRPC and SOCE in cardiac myocytes

Nearly all isoforms of the TRPC family (TRPC1–7) have been described in heart (Sabourin et al., 2011; Correll et al., 2015; Domínguez-Rodríguez et al., 2015). Their roles in cardiomyocytes have been described extensively in previous studies (Eder and Molkentin, 2011; Sabourin et al., 2011). Here, we will briefly describe their role in SOCE. Earlier studies demonstrated that a store-dependent activation involves different TRPC isoforms. Ohba et al. showed that chronic GPCRs stimulation with angiotensin II, phenylephrine or endothelin-1 evokes cardiomyocytes enlargement and enhances the expression of hypertrophic marker gene. They also demonstrated an exacerbated SOCE elicited by TG, which correlates with an enhanced TRPC1 expression. Furthermore, downregulation of TRPC1 by siRNA markedly reduces SOCE in agonists-treated cardiomyocytes (Ohba et al., 2007). Recently, TRPC1 and TRPC4 activation were associated with a passive Ca^2+^ influx induced by angiotensin II or isoproterenol, and were related to maladaptive cardiac hypertrophy since their double KO protects mice from pressure overload-induced hypertrophy and interstitial fibrosis (Camacho Londoño et al., 2015). In other study, Makarewich et al. used dominant negative mutants of TRPC4 and TRPC6 to demonstrate that only TRPC4 is sensitive to passive Ca^2+^ store depletion, while TRPC3 and TRPC6 responds to the stimulation with diacyglycerol independently of store depletion (Makarewich et al., 2014). Moreover, upregulation of TRPC3 and TRPC4 in adult ventricular cardiomyocytes correlates with the appearance of increased SOCE and pro-arrhythmic spontaneous Ca^2+^ waves (Domínguez-Rodríguez et al., 2015). Moreover, chronic treatment of cardiomyocytes with aldosterone potentiates SOCE, which is dependent of Orai1, TRPC1, TRPC4, and TRPC5 channels (Sabourin et al., 2016). Based on these findings, SOCE is likely due to the interaction of different isoforms of TRPCs, Orai1 and STIM1.

## Summary and conclusion

Substantial progress has been made in terms of understanding molecular structures and mechanisms underlying SOCE in vascular, skeletal and cardiac muscle. Compelling experimental evidences have shown that SOCE plays a central role in providing the complex spatiotemporal Ca^2+^ signals required for the regulation of diverse cellular functions such as vascular reactivity, myotubes differentiation, skeletal muscle fatigue and endurance, cardiac homeostasis and pacemaking as illustrated in Figures 1–3. SOCE also seems relevant for processes involved in muscle diseases such as cardiovascular remodeling and different myopathies. The ability of muscle cells to signal through homo- or hetero-multimeric Orai channels in combination with TRPC channels increases the diversity of Ca^2+^ signaling pathways used for the control of specific physiological and pathophysiological functions.

Consequently, given the recent advances, the singular implication of these channels in muscle physiopathology is actually considered of a major interest. Nevertheless, more studies are needed to clarify their possible use as therapeutic target for the prevention of different muscle diseases.

## Author contributions

JA-M, IM-G, AD-R, IG-C, and TS wrote different parts of the manuscript. JAR, JR, AO, and TS discuss the manuscript. TS edited the manuscript.

### Conflict of interest statement

The authors declare that the research was conducted in the absence of any commercial or financial relationships that could be construed as a potential conflict of interest.

## Abbreviations

ARC: Arachidonate-Regulated Ca^2+^
[Ca^2+^]_i_: intracellular free Ca^2+^ concentration
CCE: Capacitative Ca^2+^ Entry
CREB: cAMP Response Element Binding protein
CRAC: Ca^2+^-Release Activated Ca^2+^ Channels
DMD: Duchenne Muscular Dystrophy
EC coupling: Excitation-Contraction coupling
ER/SR: Endoplasmic/Sarcoplasmic Reticulum
GPCRs: G Protein Coupled Receptors
iPAH: idiopathic Pulmonary Arterial Hypertension
IP_3_: Inositol 1,4,5-trisphosphate
KO: knockout
LTCC: L-type Ca^2+^ Channel
NCX: Na^+^/Ca^2+^ exchanger
NFAT: Nuclear Factor of Activated T-cells
PASMCs: Pulmonary Artery Smooth Muscle Cells
PM: Plasma Membrane
PLB: Phospholamban
SERCA: Sarco/Endoplasmic Reticulum Ca^2+^ ATPase
SCID: Severe Combined Immunodeficiency
siRNAs: small interfering RNAs
SOCCs: Store-Operated Ca^2+^ Channels
SOCE: Store-Operated Ca^2+^ Entry
STIM1/2/1L: Stromal Interaction Molecule 1/2/1Large
TG: Thapsigargin
TRPC: Transient Receptor Potential-Canonical.
